# Pemphigus Vulgaris in a Sexagenarian Male: A Case Report and Review of Literature

**DOI:** 10.7759/cureus.74409

**Published:** 2024-11-25

**Authors:** Duniesky Dieguez Santiago, Sahar S Abdelmoneim, Angelica Perez Fonte, Esra Baglar, Odalys Frontela

**Affiliations:** 1 Family Medicine, Larkin Community Hospital Palm Springs Campus, Hialeah, USA; 2 Internal Medicine, Larkin Community Hospital Palm Springs Campus, Hialeah, USA; 3 Genral Internal Medicine/Cardiovascular Medicine, Assiut University Hospital, Assiut, EGY; 4 Medical School, Nova Southeastern University Dr. Kiran C. Patel College of Osteopathic Medicine, Davie, USA

**Keywords:** acantholysis, autoimmune disorder, corticosteroids, mucocutaneous, pemphigus vulgaris

## Abstract

Pemphigus vulgaris (PV) is a rare autoimmune disorder characterized by mucocutaneous blistering due to autoantibodies targeting desmoglein proteins, leading to acantholysis. This case report presents a 60-year-old Hispanic male patient with a history of hypertension who developed PV, initially presenting with pruritic scalp lesions that progressively spread to the nares, mouth, chest, neck, and inguinal region. Despite initial management with topical treatments, the lesions persisted, prompting hospitalization. Physical examination revealed characteristic skin and mucosal lesions, and a punch biopsy confirmed PV. The patient’s management involved systemic corticosteroids and supportive care, resulting in significant improvement of the lesions. This case emphasizes the challenges in recognizing and managing PV. The symptom progression from isolated scalp involvement to multiple mucocutaneous sites highlights PV’s clinical variability, which can complicate early recognition. Prompt diagnosis and a multidisciplinary approach are crucial for optimizing patient outcomes, preventing disease progression, and addressing the challenges posed by comorbid conditions.

## Introduction

Pemphigus vulgaris (PV) is an autoimmune bullous skin disorder characterized by intraepithelial blisters and edema of the skin and mucous membranes [1]. PV is caused by autoantibodies that target desmoglein 1 (Dsg1) and desmoglein 3 (Dsg3), which are components of desmosomes responsible for keratinocyte intercellular adhesions. The binding of these autoantibodies to cell-cell junctions results in the breakdown of cell-cell adhesions, resulting in acantholysis.

PV prevalence ranges from 95 per million to 145 per million persons, with an estimated annual incidence of 0.5 - 5 per 100,000 persons, and a mean age of onset between 40 and 60 years. PV is commonly associated with other autoimmune diseases such as thymoma and myasthenia gravis. A distinguishing feature of PV and other forms of pemphigus (such as pemphigus foliaceus and pemphigus vegetans) is the involvement of the oral mucosa, which presents as painful gingival erosions in 60-70% of patients [2-3]. Skin lesions usually follow the oral lesions, leading to localized or generalized involvement. Despite mortality rates remaining below 5%, PV poses a risk of long-term complications and comorbidities [3].

We present a case of PV in a 60-year-old Hispanic male patient with a medical history notable only for essential hypertension. The patient's symptom progression of initial scalp involvement extending to multiple mucocutaneous sites demonstrates the variability of PV manifestations, which can complicate early recognition. This case report highlights the importance of maintaining a high index of suspicion for PV, regardless of symptomatology, to ensure prompt recognition and intervention. The patient was informed that data concerning the case would be submitted for publication, and he provided informed consent.

## Case presentation

A 60-year-old male patient with a significant medical history of hypertension presented with a one-month history of scalp lesions characterized by yellow crusts. The patient reported initial attempts at treatment with shampoo, which provided no relief. The lesions subsequently extended to his nares, the angles of the mouth, chest, neck, and inguinal region. He described the scalp lesions as mildly pruritic and painless, without discharge. The patient denied any travel history, sick contacts, fever, chills, respiratory symptoms, chest pain, palpitations, or urinary symptoms. He also denied constitutional symptoms and any prior similar dermatological episodes. The patient's history was negative for recreational drug use, marijuana, and alcohol consumption. Family history was non-contributory. The patient’s home medications included losartan 100 mg QD and hydrochlorothiazide 12.5 mg QD. 

**Figure 1:**
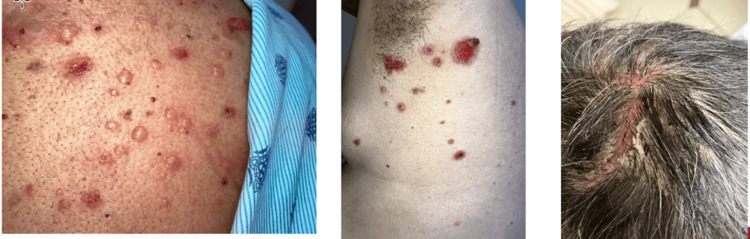
Physical examination reveals eroded skin and blistering across the limbs, torso, and scalp.

**Figure 2:**
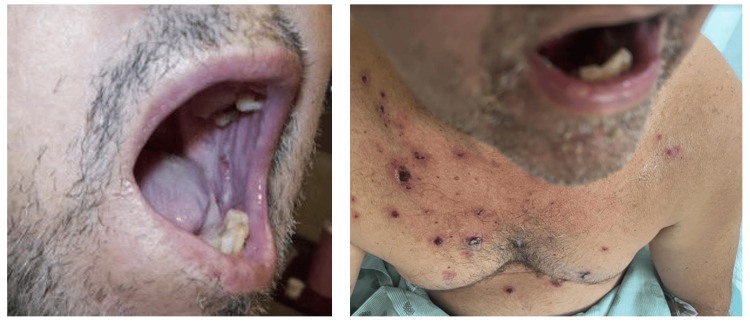
Physical examination reveals erosions on the oral mucosa and multiple lesions on the chest.

On examination, the patient was alert and oriented times three (person, time, and place). His BMI was 30.8 kg/m², and his vital signs were unremarkable, with a room air saturation of 98%. A cardiopulmonary examination revealed no abnormalities. Neck examination was negative for lymphadenopathy or thyroid gland enlargement. Abdominal examination revealed normal bowel sounds on auscultation and tenderness on light palpation of the epigastrium without rebound tenderness or guarding. Dermatological examination findings are illustrated in Figure 1 and Figure 2.

Initial management of the patient in the emergency department focused on analgesia with opioids. The patient was admitted to the hospital for further evaluation. Laboratory tests, including complete blood count (CBC), comprehensive metabolic panel (CMP), and coagulation profiles, were within normal limits. HIV testing, QuantiFERON TB testing, blood cultures, wound cultures, and urine drug screen were negative. Chest X-ray indicated a normal cardiac silhouette with no pulmonary consolidation or pleural effusion. An electrocardiogram (EKG) showed normal sinus rhythm with no ST segment or T wave abnormalities. 

A diagnosis of PV was suspected, prompting a dermatology consultation and a skin punch biopsy. While awaiting biopsy results, the patient was started on intravenous fluids and corticosteroid therapy (prednisolone 60 mg once daily). Oral lesions were managed with a steroid mouth rinse, diluted chlorhexidine mouthwash, and an oral analgesic spray administered four times daily.

Histopathological examination of the punch biopsy revealed acantholytic cells above the basal layer of the epidermis, confirming the diagnosis of PV. The patient's hospital course was uneventful, with significant improvement in oral symptoms. The treatment plan included continued prednisolone, prophylactic omeprazole 20 mg once daily to prevent gastric ulcers, calcium supplements, and alendronate 70 mg monthly to mitigate osteoporosis risk. The patient was discharged with extensive counseling on the importance of outpatient follow-up and detailed skin care instructions. At dermatology outpatient follow-up, the patient's skin lesions demonstrated full resolution with no evidence of recurrence.

## Discussion

This case contributes to the body of literature on PV, emphasizing the necessity for increased awareness and prompt recognition of the disease. Due to its unique presentation, PV poses different challenges for each patient. Individuals may exhibit minimal cutaneous involvement or significant mucocutaneous involvement, as seen in our patient, with lesions potentially restricted to the mucous membranes [1-3]. Treatment options can be limited by comorbidities. Moreover, the management of patients at the onset of the disease differs significantly from the management of those with refractory disease or multiple remissions, with the latter group often experiencing poorer health-related quality of life and treatment compliance. 

Guidelines vary in drug dosages and treatment protocols based on the severity of the disease. The treatment plan for pemphigus generally begins with prednisone at 1 mg/kg/day orally or a lower dose combined with azathioprine or mycophenolate mofetil. Rituximab may also be added. Osteoporosis and peptic ulcer prophylaxis, such as alendronate and omeprazole, are recommended. If the disease remains uncontrolled after two months, prednisone is increased to 1.5 mg/kg/day or higher intravenous doses, with continued rituximab or added azathioprine/mycophenolate mofetil, while maintaining prophylaxis. For refractory cases, high-dose prednisone or IV steroids are used, along with options like rituximab, intravenous immunoglobins (IVIG), immunoadsorption, plasmapheresis, or cyclophosphamide. Osteoporosis and ulcer prevention continue throughout treatment [1].

According to the International Pemphigus Committee, a consensus statement regarding a mutually accepted definition for PV management was developed [4]. This statement delineates minimal therapy as a regimen of ≤10 mg/day prednisolone (or equivalent) and/or minimal adjuvant therapy maintained for at least two months. Furthermore, complete remission off therapy is characterized by the absence of new or established lesions for a minimum of two months, during which the patient is not receiving any systemic therapy.

Systemic corticosteroids and immunosuppressants have long been the mainstay of pemphigus therapy, with mycophenolate mofetil and azathioprine serving as first-line steroid-sparing agents. The selection of therapeutic agents is influenced by several factors, including cost, patient comorbidities, and whether the episode is a first occurrence or a recurrence. Current evidence supports the use of rituximab as a first-line adjuvant therapy for pemphigus, demonstrating superior efficacy compared to corticosteroids alone and a reduced incidence of corticosteroid-related serious adverse events and overall mortality [1,4]. Additionally, intravenous immunoglobulin (IVIg) presents a viable therapeutic option for patients with severe and refractory PV [4].

The presence of hypertension in this case highlights the importance of selecting antihypertensive medications carefully, as some such as ACE inhibitors or beta blockers, have been reported to potentially trigger conditions like PV [5]. Clinicians managing patients with PV and hypertension must consider the possible implications of their antihypertensive regimen to avoid worsening PV symptoms. Further research is needed to clarify the relationship between PV and hypertension and to develop comprehensive management strategies for patients with both conditions.

A comparative overview of various case reports on PV in Table 1, emphasizes the diversity in clinical manifestations and treatment responses among PV patients. However, a common theme is the effective use of corticosteroids both systemic and topical, with many patients experiencing significant improvement and remission. These cases underline the importance of personalized treatment plans and regular follow-up to optimize patient outcomes and minimize complications related to treatment side effects such as osteoporosis and hyperglycemia.

**Table 1:** Comparative overview of pemphigus vulgaris (PV) case reports. Dsg3: desmoglein 3, IF, immunofluorescence.

Study	Age, gender	Clinical presentation	Diagnosis	Treatment	Follow-up
Lalremtluangi et al. (2023) [6]	60-year-old female	Multiple oral ulcers for three months, BP 160/90 mm Hg	PV based on clinical presentation	Prednisolone (40 mg) for swish and spit, triamcinolone acetonide gel, aceclofenac (100 mg) for pain	Significant improvement in three days; 90%-95% healing by second follow-up, no recurrence
Ben Lagha et al. (2005) [7]	71-year-old female	Severe mouth pain, ulcers for four months, crusted lesions on shoulders and back, depression, 7% weight loss	PV confirmed through biopsy and IF	Prednisone (40 mg/day), methotrexate (15-20 mg/week), local beclomethasone	All lesions healed in nine months, and corticosteroid therapy reduced
Miura Susanto and Uli Siahaan (2022) [8]	73-year-old female	Blisters, skin erosions, mouth ulcers, occasional fevers, reduced eating	PV based on clinical presentation	Prednisolone (70 mg/day, tapered), azathioprine, calcium, vitamin D3, IV methylprednisolone, antibiotics, insulin	Significant improvement, no recurrence three months post-treatment
Tarakji (2021) [9]	73-year-old male	Bullous lesions on gums, oral mucosa, scalp, trunk, face	PV based on biopsy and direct IF	Prednisolone (80 mg/kg/day, tapered), azathioprine	Remission at three years, side effects included osteoporosis, hyperglycemia, and hypertension due to treatment
Vega-Diez et al. (2021)[10]	68-year-old female	Pruritic, scabby, alopecic scalp plaque for six months	PV based on biopsy, IF, and positive anti-Dsg-3 antibody	Clobetasol propionate 0.05% cream	Complete healing, hair regrowth in four weeks, no recurrence over eight months

Our case report highlights the necessity for early clinical suspicion and a multidisciplinary approach in the management of PV to improve patient outcomes. Prompt referral to a dermatology specialist is crucial for early diagnosis and intervention, thereby preventing the progression to more severe disease forms and facilitating rapid recovery.

## Conclusions

Our case report contributes to the literature on pemphigus vulgaris (PV) and emphasizes the need for an early, multidisciplinary team approach to optimize patient outcomes, reduce disease morbidity, and prevent potential complications associated with delayed diagnosis and treatment. Given the chronic nature of PV and its propensity for recurrence, ongoing research is essential to further refine therapeutic strategies and improve long-term disease management. Continued collaboration between dermatology, internal medicine, and other specialties will be vital in advancing our understanding of PV and enhancing the quality of care provided to these patients.
